# The Sysmex XN‐L (XN‐350) hematology analyzer offers a compact solution for laboratories in niche diagnostics

**DOI:** 10.1111/ijlh.13339

**Published:** 2020-09-19

**Authors:** Tania A. Khartabil, Magda M. de Frankrijker, Yolanda B. de Rijke, Henk Russcher

**Affiliations:** ^1^ Department of Clinical Chemistry Erasmus MC, University Medical Center Rotterdam the Netherlands

**Keywords:** body fluid analysis, Sysmex XN‐1000, Sysmex XN‐350, Sysmex XN‐L Series, whole blood analysis

## Abstract

**Introduction:**

In 2015, Sysmex launched a new series of hematology analyzers (XN‐L Series) designed to fulfill the needs of niche laboratories in areas such as pediatrics, dialysis, neurology, and oncology while providing a compact solution. In this study, we evaluate the whole blood and body fluid modes of one of these analyzers, the XN‐350.

**Methods:**

A total of 300 residual EDTA samples were measured on the XN‐350 in whole blood mode and the XN‐1000 to evaluate method comparison, flagging sensitivity, repeatability, reproducibility, linearity, carryover, and stability. In addition, 191 samples were obtained and processed in body fluid mode which included, cerebrospinal fluid (CSF), continuous ambulatory peritoneal dialysis (CAPD), ascites, synovial, and pleural fluid to perform method comparison, repeatability, reproducibility, linearity, limit of quantitation, and carryover studies.

**Results:**

Strong agreement was shown between the XN‐350 and XN‐1000 for both whole blood and body fluid modes in results and flagging. Linearity results in both modes on the XN‐350 showed a high *R* ^2^ value (>.99). For WBC, RBC, HGB, and PLT, the carryover results were well within the predetermined criteria of ≤0.5% for whole blood and ≤0.3% for CSF. Repeatability and reproducibility were acceptable for both modes, and there were no significant deviations present in stability for whole blood. In addition, there was high agreement in all body fluid types evaluated.

**Conclusion:**

The performance of the XN‐350 is comparable to the XN‐1000 in both whole blood and body fluid modes, making it a reliable alternative to larger analyzers for smaller, niche laboratories.

## 1 INTRODUCTION

Since the beginning of the automation of laboratory processes in the 1950s, blood cell analysis has evolved from labor intensive, manual procedures heavily depending on the microscopic determination to high flow‐through, automatic systems.^1^, ^2^ Currently, it is not uncommon for large routine laboratories to process thousands of samples a day providing millions of complete blood count tests on a yearly basis. These laboratories make use of multiple, large high throughput hematology analyzers as part of an automated laboratory transport system, however, not all laboratories have the financial ability, access, or need to use these high flow‐through automated systems.^3^ This causes many smaller clinics and laboratories to send out their samples to larger centers, which takes additional time to obtain patient results. To address this issue, in 2015 Sysmex launched a new series of hematology analyzers, the XN‐L Series. This series contains the XN‐350, XN‐450, and XN‐550 analyzers. These analyzers are based on Sysmex XN‐Series technology, but especially designed to fulfill the needs for small to medium laboratories, emergency laboratories, satellite laboratories, specialty laboratories, and laboratories and also as a backup system for routine analyzers.

This study is meant to emphasize the performance of the XN‐350 to serve smaller, specialized laboratories as supposed to its counterpart, the XN‐1000, that better serves larger scale centers. The reference for the Sysmex XN‐Series is the XN‐1000 as a high throughput automated hematology analyzer and was first evaluated in 2012 for both whole blood mode and body fluid mode.^4^, ^5^, ^6^ Thereafter, its accuracy and precision have been described for various sample types and medical conditions and compared with other leading hematology analyzers.^6^, ^7^, ^8^, ^9^, ^10^ A subset of the XN technology and measurement channels are also used in XN‐L series: A complete blood count (CBC) with a 6‐part white blood cell (WBC) differential, where immature granulocytes (IG) are counted in addition to the 5‐part differential. Optionally, the XN‐L can be extended with a RET channel and a body fluid mode (XN‐BF). The XN‐350 offers single sample analysis in open mode only.

To date the XN‐L series was evaluated in several studies, however, no combined studies can be found since only whole blood mode or body fluid mode was evaluated. A good correlation was generally observed for all parameters, except for basophils. Tailor et al. focused especially on the different platelet counting methods (impedance and optical) of the XN‐550 and observed reliable results especially in the low counts (<40 × 10^9^/L) when triggers for preventive platelet transfusions are established.^11^ The whole blood module of the XN‐350 was evaluated for determination of routine hematology parameters in hematopoietic progenitor cell apheresis products. This study gave reliable results, but the RBC counts were overestimated, possibly due to interference of WBC in the impedance counting of RBCs.^12^ The XN‐350 body fluid mode has been evaluated in two studies. The first study established a cut‐off for the detection of high fluorescence cells in pleural and ascites fluids as an indicator for malignancy^13^ and the second, more recent study evaluated the analytical performance of the body fluid mode with two other systems, the UniCel DxH800 and the UF‐5000. The XN‐350 showed the most comparable results to those of manual differential counting.^14^

However, there has yet to be a comprehensive study of the Sysmex XN‐350 hematology analyzer and its performance for both whole blood and body fluid analysis combined. In this study, we evaluated the XN‐L (XN‐350) by comparing all common whole blood and body fluid parameters between the XN‐350 and the XN‐1000. Furthermore, for both whole blood and body fluid analysis we performed an analytical evaluation for repeatability, reproducibility, linearity, carryover, lower limit of detection as well as flagging performances and stability for whole blood analysis.

## 2 MATERIAL AND METHODS

### 2.1 Sample collection

Only residual samples were included in this study as approved by the Medical Ethics Review Committee at Erasmus Medical Center, study number MEC‐2018‐1278. For whole blood samples, a total of 300 residual samples were collected consisting of 50 samples from healthy individuals with results within the reference ranges established by Erasmus MC and 250 abnormal samples based on pathology or cell count including immature granulocytes, abnormal lymphocytes, atypical lymphocytes and blasts and sample results outside the established reference ranges. For body fluids, 191 samples were also used in this study which included pleural, peritoneal (ascites), cerebrospinal (CSF), peritoneal dialysate (CAPD), and synovial fluids. All samples were collected in EDTA tubes (Becton Dickinson VACUTAINER disodium EDTA), with the exception of CSF samples, which were collected without additives and submitted to the Erasmus MC Department of Clinical Chemistry for routine testing. Samples with a volume <500 μL were excluded, due to small sample volume. No pediatric samples were included in the study.

### 2.2 Method comparison

Method comparison studies were performed to assess the performance of the XN‐L series (XN‐350) analyzer compared with the XN‐1000 analyzer for whole blood and body fluids. For whole blood, a total of 300 whole blood samples were included. All samples were run within two hours after venipuncture and within two hours of both runs on each analyzer. Samples covered clinical decision levels and the full reportable measuring ranges of the XN‐Series analyzers including immature granulocytes, abnormal lymphocytes, atypical lymphocytes, and blasts. Samples were processed on the XN‐350 in the whole blood mode in the CBC + DIFF + RET channel profile and measured on the XN‐1000 in the full channel profile (CBC + DIFF + RET + PLT‐F + WPC). All samples were run in duplicate and the comparisons were performed with the average results. The following parameters were measured on both systems; white blood cells (WBC), red blood cells (RBC), hemoglobin (HGB), hematocrit (HCT), mean corpuscular volume (MCV), mean corpuscular hemoglobin (MCH), mean corpuscular hemoglobin concentration (MCHC), red cell distribution width (RDW‐SD, RDW‐CV), platelets (PLT), platelet distribution width (PDW), mean platelet volume (MPV), plateletcrit (PCT), % and # for neutrophils (NEUT), lymphocytes (LYMPH), monocytes (MONO), eosinophils (EO), basophils (BASO), immature granulocytes (IG), reticulocytes (RET) as well as reticulocyte hemoglobin content (RET‐He). For the platelet parameter specifically, the XN‐350 does not have the capability of measuring PLT‐F (platelet‐fluorescence) so instead, PLT‐I (platelet‐impedance) and PLT‐O (platelet‐optical) were evaluated in this study. The method comparison study was performed in accordance with CLSI_H20‐A2. The nucleated red blood cells (NRBC) parameter was not included in this evaluation because NRBC% and NRBC# are only research parameters on XN‐L analyzers because these parameters are quantitated in WDF channel and not in WNR as in XN‐1000.

To compare the performance of the body fluid mode in the XN‐350 to the XN‐1000, BF samples were collected consisting of 53 CSF, 53 ascites, 25 synovial, 29 CAPD, and 31 pleural fluid for a total of 191 samples. Samples were processed in duplicate on both analyzers and the average WBC, RBC, polymorphonuclear leukocyte count (#PMN), and mononuclear leukocyte count (#MN) results were compared.

### 2.3 Flagging performance

The overall flagging performance of XN‐350 vs XN‐1000 was evaluated for immature granulocytes (IGs), blasts, abnormal lymphocytes, atypical lymphocytes, and left shift on the same 300 samples used for method comparison. Smears were made on each sample and a manual differential was performed on the CellaVision DM‐96 digital cell morphology system (CellaVision AB). Following the DM‐96 results, smears were then reviewed by trained medical technologists for the final determination of all cell types including abnormal cells present such as bands, blasts, atypical lymphocytes, and immature granulocytes. Various medical technologists completed a proficiency examination to minimize variability in smear review results. An abnormal manual differential was defined according to the criteria for action following automated CBC and differential WBC differential analysis as suggested by the international consensus group for hematology of the International Society for Laboratory Hematology (ISLH).^15^ In short: immature granulocytes ≥1%, blasts ≥1% and/or abnormal lymphocytes ≥5% (which included plasma cells), atypical Lymphocytes ≥5% and left shift was band ≥5% and/or IG ≥1%. The sensitivity, specificity, positive predictive value (PPV), negative predictive value (NPV), and efficiency were calculated for each individual flag that relates to the morphology or presence of abnormal cells.

### 2.4 Repeatability and reproducibility

Repeatability studies were performed using residual EDTA whole blood samples covering clinical decision levels and the upper and lower limit of the analytical measuring range. Twenty replicates of each sample were tested in the whole blood manual mode on the XN‐350. The mean, standard deviation (SD), and coefficient of variation (CV) were calculated for each sample. After processing the samples in whole blood mode, the same samples were diluted with Cellpack DCL (1:7) and measured in the pre‐diluted mode.

To determine repeatability of body fluids, one sample of each type of body fluid was processed ten times consecutively to determine if the variability in results exceeded the acceptance criteria of being within 20% for any particular body fluid type specifically. Each sample was divided into two tubes and processed five times each for a total of ten runs. In addition, three different concentrations of CSF were included with a final concentration of WBC ≦4/μL (CSF4), 10/μL (CSF10) and 50/μL (CSF50).

The reproducibility was performed by using XN CHECK levels 1, 2, and 3 for the whole blood mode and XN CHECK BF levels 1 and 2 for the body fluid mode. All levels were processed twice a day in triplicate across a five‐day period in order to provide additional reproducibility data accounting for time and day variability. Studies were performed in accordance with the recommendations in CLSI_EP05‐A3.

### 2.5 Linearity

Seven serial dilutions of known, high concentration of whole blood samples in EDTA were prepared with the lowest dilution reaching the limit of quantitation (LoQ) established by Sysmex. The samples were run on the XN‐350 in the CBC + DIFF + RET mode in duplicate and the WBC, RBC, HGB, and PLT parameters were evaluated. The LoQ for the XN‐350 is defined at 0.03 × 10^3^/µL (WBC), 0.01 × 10^6^/µL (RBC), 0.1 g/dL (HGB), and 5 × 10^3^/µL (PLT‐I/PLT‐O) in the XN‐350 Instructions for Use. Both the CLSI_H26‐A2, 201 and NCCLS_EP6‐A, 2012 guidelines were followed in this linearity study.

In addition, linearity for WBC and RBC in body fluid mode was determined by selecting a CSF sample with a high concentration of cells to be serially diluted in PBS buffer. Samples with approximately 500 WBC/μL and >1000 RBC/μL were diluted 1:10, 1:20, 1:50, and 1:200 with PBS and each of these dilutions was measured 5 times consecutively on the XN‐350.

### 2.6 Carryover

Carryover was evaluated by measuring whole blood samples with high target values (HTV) for WBC, RBC, HGB, and PLT counts three consecutive times (H1, H2, and H3) followed immediately by testing samples with low target values (LTV) around clinical decision levels consecutively, three times (B1, B2, and B3). Carryover effect was calculated for each parameter using the Broughton method, [(B1‐B3)/(H3‐B3)] × 100%. In addition, carryover was assessed for body fluids on CSF samples containing high and low WBC and RBC counts. The Sysmex standard is a carry‐over ratio coefficient <0.3% or a maximum difference of 1 cell/μL between B1 and B3. The carryover study design was performed in accordance with CLSI_H26‐A2.

### 2.7 Stability

Stability of whole blood was determined using residual samples from five normal individuals and five patients with abnormalities. The 5 abnormal blood samples contained one of the following parameters with a value well above or below the reference value, RBC, WBC, HGB, PLT, HCT, and RDW‐CV. The other 5 normal blood samples had either normal values within the reference intervals or values that fell slightly below or above the reference value, but were not regarded as abnormal. All samples were run on the XN‐350 (time point 0 hour) and subsequently aliquoted in two sets of six aliquots. One set was stored at room temperature (RT) and the second at 4°C. It was determined that 4°C would be used for stability based on ICSH guidelines.^16^ One aliquot of both sets was measured on the XN‐350 at time point 4, 8, 12, 24, 48, and 72 hours, respectively. For each time point and temperature setting, the average value of each CBC parameter (all 10 samples) was measured and compared to time point 0 hour. The acceptable change limit (ACL) was then calculated via $\sqrt{\left[{\left(2.77\;\text{CVa}\right)}^{2}+{\left(0.5\;\text{CVb}\right)}^{2}\right]}$ and compared to the corresponding storage times according to ISO 5725‐6.^17^

### 2.8 Limit of quantitation

Due to the potentially low WBC and RBC values in body fluid samples, a limit of quantitation needed to be determined on the XN‐350. Six CSF samples were selected with very low concentrations (1 WBC/μL and 10 RBC/μL), and samples were diluted to achieve such results if they were not found naturally. Each sample was processed 5 times in order to obtain 30 analytical results in total. The limit of quantitation (LoQ) was determined to be at the concentration of the samples at which the coefficient of variation (CV) of <20% is achieved. The study was performed in accordance with CLSI EP17‐A2, and the CV was calculated for each sample separately.

### 2.9 Statistical analysis

For all statistical analysis, the absolute cell counts were used for all measurements. Data analysis was performed by using Analyse‐it for Microsoft Excel version 2.21 and Microsoft Excel 2013. Passing‐Bablok regression analysis and Bland‐Altman bias plots were used for method comparison and linearity studies. In addition, the coefficient of determination (*R* ^2^) was calculated for linearity to determine variation across the measured range. Statistical significance was based on the 95% confidence intervals (CI). A significant proportional or constant bias was noted when the 95% CI of the slope did not encompass 1, and the 95% CI of the mean difference, limits of agreement (LoA) or intercept did not encompass 0, respectively. Spearman's correlation coefficient (*r*_s_ value) was used in the method comparison analysis. Acceptance criteria are according to the manufacturer's criteria in the Sysmex Instructions for Use.

## 3 RESULTS

### 3.1 Method comparison

**Figure 1:**
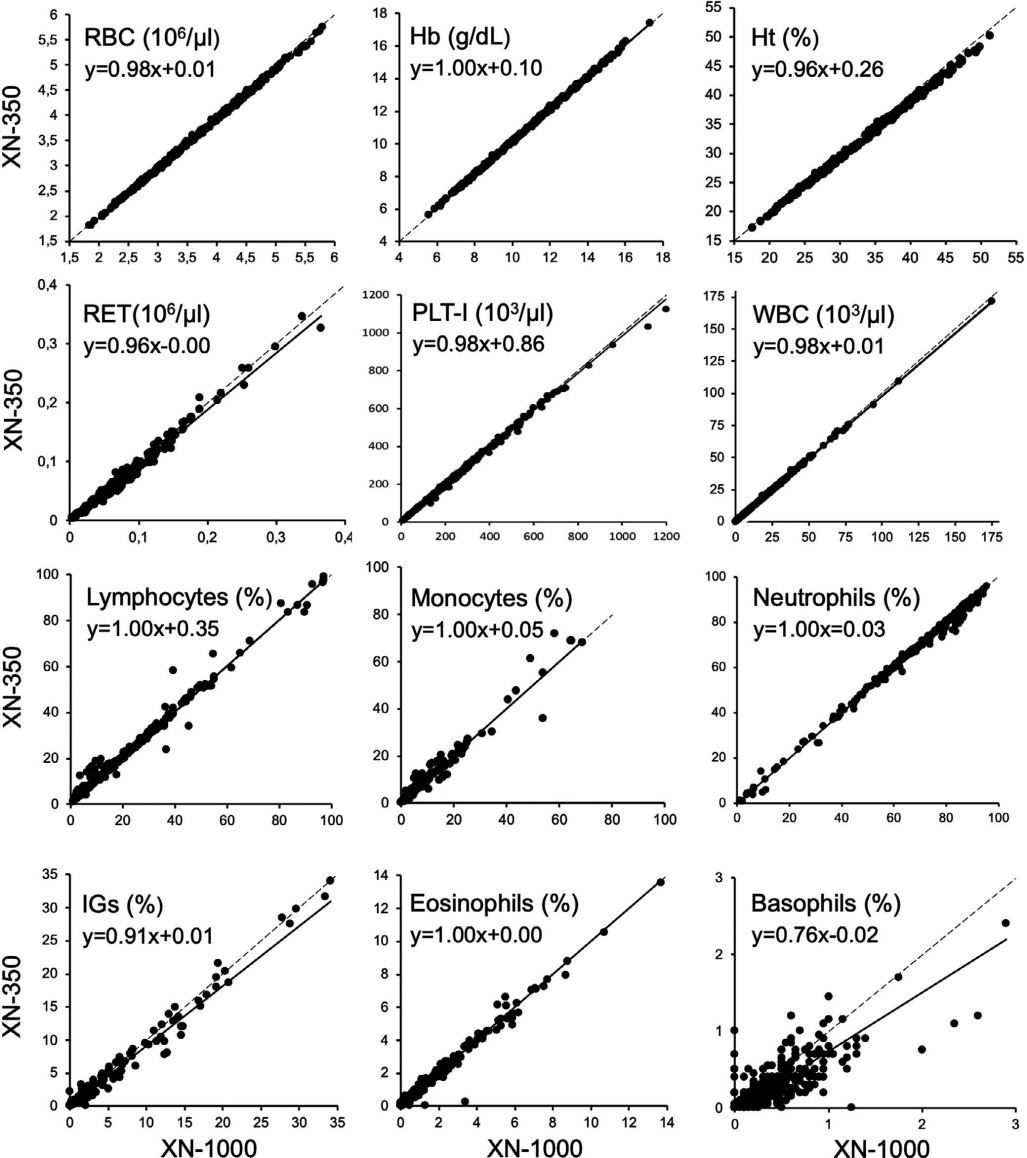
Passing‐Bablok Regression Comparison Between XN‐350 and XN‐1000

For the evaluation of whole blood, a total number of 300 residual EDTA whole blood samples were analyzed on both the XN‐350 and the XN‐1000. Table 1 shows the correlation and the estimated bias for the 22 parameters measured and Figure 1 shows the Passing‐Bablok plots for all directly measured parameters. The plots for the remaining calculated are in Figure S1. The results of the linear regression and the bias analysis between the XN‐350 and the XN‐1000 indicated that all applicable parameters met the acceptance criteria as established by the manufacturer. Out of the 22 parameters measured, 21 parameters have an excellent Spearman's *r*_s_ correlation coefficient of ≥.90. The basophil count seems less accurate with an r_s_ of 0.769.

**Table 1:** Method Comparison of XN‐350 Compared to XN‐1000 in Whole Blood Mode

Measurand	Unit	n*	Spearman's *r*_s_	Range (median)	Bland‐Altman statistics		Passing‐Bablok regression	
					Mean diff. (95% CI)	95% LoA	Slope (95% CI)	Mean diff. (95% CI)
WBC	10^3^/μL	300	1.00	0.11 to 175.4 (14.0)	−*0.40 (−0.46 to −0.34)*	−1.48 to 0.68	*0.98 (0.97 to 0.98)*	0.01 (−0.01 to 0.03)
RBC	10^6^/μL	300	0.999	1.84 to 5.80 (3.62)	−*0.07 (−0.07 to −0.06)*	−0.13 to 0.00	*0.98 (0.98 to 0.98)*	0.01 (−0.01 to 0.02)
HGB	g/dL	300	1.00	5.55 to 17.3 (10.6)	*0.08 (0.07 to 0.09)*	−0.04 to 0.20	1.00 (1.00 to 1.00)	*0.10 (0.10 to 0.10)*
HCT	%	300	0.999	17.55 to 51.4 (34.1)	−*1.00 (−1.05 to −0.96)*	−*1.80 to −0.21*	*0.96 (0.96 to 0.97)*	*0.26 (0.12 to 0.39)*
MCV	fL	300	0.995	63.1 to 111.6 (92.7)	−*1.07 (−1.12 to −1.01)*	−*2.00 to −0.14*	*1.02 (1.01 to 1.03)*	−*3.05 (−3.89 to −2.12)*
MCH	Pg	300	0.978	18.75 to 36.55 (29.8)	*0.78 (0.75 to 0.81)*	*0.25 to 1.30*	1.02 (1.00 to 1.03)	0.35 (−0.19 to 0.80)
MCHC	g/dL	300	0.958	27.4 to 36.3 (31.98)	*1.22 (1.19 to 1.26)*	*0.60 to 1.85*	1.02 (1.00 to 1.06)	0.53 (−0.54 to 1.25)
PLT‐I	10^3^/μL	300	0.998	7.50 to 1199.0 (143.50)	−*5.37 (−6.55 to −4.20)*	−25.69 to 14.93	*0.98 (0.98 to 0.99)*	0.86 (−0.03 to 1.60)
PLT‐O	10^3^/μL	300	0.995	6.00 to 1253.0 (135.5)	−*5.48 (−7.84 to −3.11)*	−46.24 to 39.33	*0.97 (0.96 to 0.98)*	*4.46 (3.05 to 6.21)*
RDW‐SD	fL	300	0.998	34.3 to 86.75 (49.13)	−*0.46 (−0.52 to −0.41)*	−1.46 to 0.54	1.00 (0.99 to 1.00)	−0.25 (−0.45 to 0.09)
RDW‐CV	%	300	0.999	11.25 to 25.2 (14.6)	−*0.09 (−0.11 to −0.08)*	−0.33 to 0.14	1.00 (1.00 to 1.00)	−*0.10 (−0.10 to −0.10)*
PDW	fL	299	0.957	8.2 to 23.55 (12.55)	−*0.84 (−0.91 to −0.76)*	−2.13 to 0.45	*0.94 (0.91 to 0.96)*	−0.08 (−0.37 to 0.23)
MPV	fL	299	0.972	8.55 to 14.6 (10.9)	−*0.35 (−0.38 to −0.32)*	−0.81 to 0.12	*0.96 (0.93 to 0.98)*	0.10 (−0.19 to 0.33)
PCT	%	300	0.998	0.01 to 1.19 (0.25)	−*0.01 (−0.02 to −0.01)*	−0.04 to 0.01	*0.94 (0.94 to 0.95)*	0.00 (0.00 to 0.00)
NEUT#	10^3^/μL	300	1.000	0.01 to 83.9 (7.90)	−*0.42 (−0.49 to −0.34)*	−1.67 to 0.83	*0.97 (0.97 to 0.98)*	−0.01 (−0.02 to 0.01)
LYMPH#	10^3^/μL	300	0.979	0.03 to 107.9 (1.61)	0.03 (−0.06 to 0.11)	−1.43 to 1.48	*0.98 (0.98 to 0.99)*	*0.03 (0.01 to 0.04)*
MONO#	10^3^/μL	300	0.981	0 to 121 (0.91)	0.03 (−0.05 to 0.10)	−1.24 to 1.29	1.00 (0.99 to 1.02)	−0.01 (−0.02 to 0.00)
EO#	10^3^/μL	300	0.984	0 to 1.93 (0.08)	0.00 (0.00 to 0.00)	−0.04 to 0.04	*0.97 (0.96 to 0.98)*	0.00 (0.00 to 0.00)
BASO#	10^3^/μL	300	0.769	0 to 1.19 (0.05)	−*0.03 (−0.03 to −0.02)*	−0.12 to 0.08	*0.55 (0.50 to 0.60)*	0.00 (0.00 to 0.01)
IG#	10^3^/μL	300	0.979	0 to 23.65 (0.11)	−*0.10 (−0.14 to −0.07)*	−0.71 to 0.51	*0.88 (0.86 to 0.90)*	0.00 (0.00 to 0.00)
RET#	10^6^/μL	300	0.986	0 to 0.36 (0.07)	0.00 (0.00 to 0.00)	−0.02 to 0.01	*0.96 (0.94 to 0.98)*	0.00 (−0.01 to 0.00)
RET‐He	Pg	300	0.941	20.2 to 41.4 (32.2)	−*0.98 (−1.09 to −0.86)*	−2.96 to 1.01	*1.13 (1.11 to 1.16)*	−*5.05 (−5.95 to −4.18)*

Note

Bland‐Altman (mean difference and limits of agreement) statistics and Passing‐Bablok regression (slope and intercept) are shown. Each sample was processed in duplicate and the average values for both runs were analyzed. Values in *italic* denote a statistically significant difference. The median reflects that of the comparative method (ie XN‐1000). n, sample number; *r*_s_, Spearman's correlation coefficient; 95% CI, 95% confidence interval; Mean diff., mean difference; LoA, upper and lower limits of agreement.

The agreement results for WBC, RBC, PMN, and MN in body fluids are presented in Table 2 and Passing‐Bablok plots for WBC and RBC are shown in Figure S2. Every parameter except RBC values for CSF samples had an r_s_ value of at least 0.90 and no clinically significant bias. The *r*_s_ for RBC in the CSF samples was 0.857, however, 52 of the 53 samples included had an RBC value of 0.0. The slope of the trend lines for all parameters was acceptable within 1.0 ± 0.30. The statistically significant differences in the Bland‐Altman statistics and Passing‐Bablok regression analysis for both the whole blood and the body fluid mode were small and clinically irrelevant.

**Table 2:** Method comparison of XN‐350 vs XN‐1000 in Body fluid mode

Body fluid type	Measurand	Unit	n	Spearman's *r*_s_	Range (median)	Bland‐Altman statistics		Passing‐Bablok regression	
						Mean Diff. (95% CI)	95% LoA	Slope (95% CI)	Intercept (95% CI)
**CSF**	WBC	10^3^/μL	53	0.989	0.00 to 3.43 (0.01)	0.00 (0.00 to 0.01)	−0.03 to 0.04	1.01 (1.00 to 1.04)	0.00 (0.00 to 0.00)
	RBC	10^6^/μL	53	0.857	0.00 to 0.15 (0.00)	0.00 (0.00 to 0.00)	0.00 to 0.00	0.99 (0.98 to 1.00)	0.00 (0.00 to 0.00)
	MN#	10^3^/μL	53	0.979	0.00 to 1.41 (0.01)	0.00 (0.00 to 0.01)	−0.01 to 0.02	*1.03 (1.01 to 1.05)*	0.00 (0.00 to 0.00)
	PMN#	10^3^/μL	53	0.911	0.00 to 2.72 (0.00)	0.00 (0.00 to 0.01)	−0.02 to 0.03	1.03 (1.00 to 1.04)	0.00 (0.00 to 0.00)
**Ascites**	WBC	10^3^/μL	53	0.998	0.05 to 19.97 (0.27)	0.01 (−0.03 to 0.05)	−0.27 to 0.28	1.00 (0.99 to 1.02)	0.00 (0.00 to 0.00)
	RBC	10^6^/μL	53	0.957	0.00 to 0.53 (0.00)	0.00 (0.00 to 0.00)	−0.01 to 0.00	1.00 (0.99 to 1.00)	0.00 (0.00 to 0.00)
	MN#	10^3^/μL	53	0.997	0.05 to 5.26 (0.24)	0.01 (−0.01 to 0.02)	−0.12 to 0.13	1.01 (0.99 to 1.02)	0.00 (0.00 to 0.00)
	PMN#	10^3^/μL	53	0.992	0.00 to 18.74 (0.03)	0.00 (−0.05 to 0.05)	−0.33 to 0.33	1.00 (0.98 to 1.02)	0.00 (0.00 to 0.00)
**Synovial**	WBC	10^3^/μL	25	0.998	0.01 to 36.47 (2.96)	*0.43 (0.19 to 0.67)*	−0.70 to 1.55	*1.08 (1.03 to 1.12)*	−0.02 (−0.09 to 0.02)
	RBC	10^6^/μL	25	0.992	0.00 to 0.12 (0.00)	0.00 (0.00 to 0.00)	0.00 to 0.00	1.00 (1.00 to 1.06)	0.00 (0.00 to 0.00)
	MN#	10^3^/μL	25	0.997	0.01 to 6.03 (1.36)	*0.16 (0.08 to 0.25)*	−0.25 to 0.57	*1.08 (1.04 to 1.11)*	0.00 (−0.04 to 0.01)
	PMN#	10^3^/μL	25	0.999	0.00 to 31.13 (0.33)	*0.27 (0.08 to 0.46)*	−0.63 to 1.17	*1.07 (1.03 to 1.11)*	0.00 (−0.02 to 0.00)
**CAPD**	WBC	10^3^/μL	29	0.999	0.00 to 5.43 (0.12)	−0.01 (−0.03 to 0.00)	−0.10 to 0.07	0.99 (0.97 to 1.00)	0.00 (0.00 to 0.00)
	RBC	10^6^/μL	29	*0.998*	0.00 to 1.00 (0.00)	−0.03 (−0.11 to 0.04)	−0.40 to 0.33	1.00 (N/A)	0.00 (N/A)
	MN#	10^3^/μL	28	0.995	0.00 to 0.82 (0.09)	0.00 (0.00 to 0.00)	−0.02 to 0.02	0.98 (0.96 to 1.00)	0.00 (0.00 to 0.00)
	PMN#	10^3^/μL	28	0.987	0.00 to 4.61 (0.02)	−0.01 (−0.02 to 0.00)	−0.09 to 0.06	0.98 (0.95 to 1.00)	0.00 (0.00 to 0.00)
**Pleural**	WBC	10^3^/μL	31	0.999	0.16 to 5.39 (1.34)	*0.02 (0.01 to 0.04)*	−0.06 to 0.11	1.01 (0.99 to 1.03)	0.00 (−0.01 to 0.02)
	RBC	10^6^/μL	30	0.998	0.00 to 1.33 (0.01)	0.00 (0.00 to 0.00)	0.00 to 0.00	1.00 (1.00 to 1.00)	0.00 (0.00 to 0.00)
	MN#	10^3^/μL	31	0.998	0.08 to 5.31 (0.90)	*0.03 (0.01 to 0.04)*	−0.05 to 0.10	1.02 (1.00 to 1.03)	0.00 (0.00 to 0.01)
	PMN#	10^3^/μL	31	0.994	0.01 to 1.83 (0.24)	0.00 (−0.01 to 0.00)	−0.06 to 0.05	0.99 (0.94 to 1.00)	0.00 (0.00 to 0.01)

Note

Bland‐Altman (mean difference and limits of agreement) statistics and Passing‐Bablok regression (slope and intercept) are shown. Each sample was processed in duplicate and the average values for both runs were analyzed. Values in *italic* denote a statistically significant difference. The median reflects that of the comparative method (ie XN‐1000). n, sample number; *r*_s_, Spearman's correlation coefficient; 95% CI, 95% confidence interval; Mean diff., mean difference; LoA, upper and lower limits of agreement; N/A, not applicable.

### 3.2 Flagging sensitivity

Flagging sensitivity, specificity, NPV, and PPV are included in Table 3. Both the XN‐350 and the XN‐1000 had similar sensitivity and specificity for the abnormal cell types measured. For each system flag, both analyzers flagged around the same amount with the exception of left shift that flagged much more frequently on the XN‐350 (110/300) compared to the XN‐1000 (66/300). Moreover, both systems have indicated the correct flag at about the same frequency as determined by combining true positive and true negative results. For atypical lymphocytes, the sensitivity was really low for the XN‐350 and XN‐1000 (8.8% and 2.9%), but had the highest specificity (96.2% and 94.7%) compared to other flags measured.

**Table 3:** Flagging performance on XN‐350 and XN‐1000

Flag (samples flagged/total samples)	True Positive	False Positive	True Negative	False Negative	Sensitivity	Specificity	PPV	NPV	Efficiency
					(%)	(%)	(%)	(%)	(%)
IG Present?									
XN‐350 (137/300)	68	69	141	22	75.6	67.1	49.6	86.5	69.7
XN‐1000 (147/300)	72	79	131	18	80.0	62.4	47.7	87.9	67.7
Blast?/Abn Lympho?									
XN‐350 (69/300)	19	50	229	2	90.5	82.1	27.5	99.1	82.7
XN‐1000 (64/300)	19	36	243	2	90.5	87.1	34.6	99.2	87.3
Atyp Lympho?									
XN‐350 (13/300)	3	10	256	31	8.8	96.2	23.1	89.2	86.3
XN‐1000 (15/300)	1	14	252	33	2.9	94.7	6.7	88.4	84.3
Left Shift?									
XN‐350 (110/300)	69	41	161	29	70.4	79.7	62.7	84.7	76.7
XN‐1000 (66/300)	51	15	187	47	52.0	92.6	77.3	79.9	79.3

Abbreviations Abn Lympho, abnormal lymphocytes; Atyp Lympho, atypical lymphocytes; TP, true positive; FP, false positive; TN, true negative; FN, false negative; PPV, positive predictive value; NPV, negative predictive value.

### 3.3 Repeatability and reproducibility results

Table S1 shows the calculated coefficient of variation (%CV) and the predefined manufacturer's specifications for within‐run precision. All parameters measured in the whole blood mode met the specifications for precision set by Sysmex. Similarly, pre‐diluted samples processed in the pre‐dilution mode also met the required specifications for precision set by Sysmex. In addition, all three levels of controls were processed twice a day over the course of five days to account for day to day variability. Table S2 shows the reproducibility results for all three control levels and all parameters measured did not exceed the %CV set by manufacturer. For CBC parameters, all parameters showed <5% CV with the exception of platelets for Level 1, however, in whole blood the PLT variability was within range. Very high %CV values were noted for parameters such as eosinophils and basophils due to the low percentages present in the control material.

Reproducibility results for body fluids parameters are shown in Table S3, and the results were within the acceptance criteria established by Sysmex for every parameter. Repeatability results are shown for all body fluid types as in Table S4A‐G. Some exceeded the acceptance criteria established by the manufacturer of having a CV <20%, such as the synovial fluid and CSF10. The highest CV calculated for WBC was still only 22.5%. #PMN and #MN had very high %CV for these parameters as it was quite difficult to collect samples with a high enough number of cells. These numbers were very close to 0, therefore, the slightest variability caused the large %CV. Overall for body fluid mode, the reproducibility of the XN‐350 meets the requirements set by Sysmex (Table S5).

### 3.4 Linearity results

For both whole blood and body fluid analysis, the XN‐350 demonstrated to be linear from lower limit to upper limit and remained within the allowable maximum % diff for each interval. Table 4 shows whole blood results for WBC, RBC, HGB and PLT and body fluid linearity results for WBC and RBC. The R^2^ values for all parameters measured are above 0.99 with minimal bias and no outliers.

**Table 4:** Linearity of the XN‐350 in Whole Blood and Body Fluid Modes

Parameter	Range	*R* ^2^	Bias	Regression Line
WBC (10^3^/μL)	0.03‐418.70	1.00	0.07	*y* = 418.21*x* + 0.07
RBC (10^6^/μL)	0.00‐8.61	.999	−0.02	*y* = 8.54*x* − 0.02
HGB (g/dL)	0.00‐21.75	.999	−0.08	*y* = 13.74*x* − 0.08
PLT (10^3^/μL)	1‐971	.999	−6.01	*y* = 965.03*x* − 6.01
WBC‐BF (10^3^/μL)	0.00‐0.54	.999	0.00	*y* = 1.09*x* − 0.00
RBC‐BF (10^6^/μL)	0.00‐0.02	.998	0.00	*y* = 0.035*x* − 0.00

### 3.5 Carryover results

Table S6 shows the recommended criteria for the high and low target values of the samples selected to evaluate carryover as well as the corresponding carryover ratio. The high RBC and HGB samples had to be manually manipulated in order to reach the established threshold. These samples were spun down in a centrifuge and plasma was removed. The carryover ratio for WBC, RBC, HGB, and PLT was 0.03%, 0.29%, 0.00%, and 0.20%, respectively, and stayed within the manufacturer's specifications (≤1%).

For body fluids, Table S7 shows the HTV sample had a WBC concentration of 20.440 × 10^3^/µL and the LTV sample of 0.001 × 10^3^/µL. For RBC, the HTV was 20 × 10^3^/µL and the LTV sample was 1 × 10^3^/µL. For WBC and RBC, the carryover calculated was well below the acceptable criteria. Using the Broughton method, this showed that the carryover is less than 0.3% and is therefore negligible for WBC in CSF.

### 3.6 Stability results

Seven parameters were measured in aliquots of whole blood, stored at 4°C and RT, of the same sample at the initial time point (0 hour) and time point 4, 8, 12, 24, 48, and 72 hours. Table 5 shows the differences between the values measured at the initial time point and the time points of interest for both temperature settings. Both WBC and RBC parameters in whole blood remain stable until 72 hours after obtaining the blood samples. Hemoglobin levels did not exceed the ACL when stored at 4°C and are stable up to 72 hours after sampling. However, when stored at RT, the change in HGB is higher than the ACL indicating a stable storage time of 12 hours instead of the 72 hours indicated by Sysmex. The findings indicate that the HCT levels are stable until 72 hours (4°C) and 12 hours (RT), while the MCV levels are only stable for 4 hours after blood samples independent of the storage temperature. PLT counts are stable for 72 hours (4°C) and 12 hours (RT) and RDW remains stable for 72 hours (4°C) and 24 hours (RT).

**Table 5:** Whole blood stability on the XN‐350 at 4°C and room temperature

Parameter	Range	Temp. [°C]	ΔX 4 h [%]	ΔX 8 h [%]	ΔX 12 h [%]	ΔX 24 h [%]	ΔX 48 h [%]	ΔX 72 h [%]	ACL [%]	Stable until [h]
WBC (10^3^/μL)	0.36 to 31.6	4 RT	−0.1 0.4	2.1 1.9	2.5 2.0	2.2 1.2	3.0 −1.5	2.8 −4.9	6.3	72 72
RBC (10^6^/μL)	2.03 to 4.88	4 RT	2.1 2.1	2.2 2.3	2.5 2.4	2.2 2.2	2.0 2.3	1.5 2.8	3.1	72 72
HGB (g/dL)	5.96 to 13.7	4 RT	2.0 1.4	1.7 2.1	1.9 2.0	1.9 2.9*	1.8 2.2	1.2 1.7	2.8	72 12
HCT (%)	18.3 to 42.5	4 RT	−1.9 −1.9	−2.1 −0.9	−1.6 −0.5	−1.5 3.2*	−0.1 8.5*	0.4 15.3*	3.0	72 12
MCV (fL)	87.1 to 108.1	4 RT	−3.6* −3.9*	−3.8* −3.2	−3.8* −2.8	−3.3 −0.1	−1.7 6.1*	−0.6 12.2*	3.3	<4 <4
RDW‐CV (%)	12.6 to 22.9	4 RT	−1.7 −0.8	−1.7 −1.0	−2.0 −0.7	−2.5 1.1	−3.0 5.8*	−3.3 9.2*	5.1	72 24
PLT (10^3^/μL)	18.0 to 421.0	4 RT	−7.2 −3.5	−5.5 −6.2	−9.9 −9.8*	−8.2 −14.7*	−7.9 −23.5*	−7.8 −23.8*	10.5	72 12

Note

Relative average deviations for WBC, RBC, HGB, HCT, MCV, RDW, and PLT between baseline and the storage times (4 h up to 72 h) at 4°C and room temperature in whole blood mode. ΔX, time difference; Temp., temperature; RT, room temperature; ACL, Acceptable Change Limit according to ISO 5725‐6; ACL = SQRT[(2.77 CVa)^2^ + (0.5 CVb)^2^]. Values exceeding the ACL are marked with an asterisk (*).

### 3.7 Limit of quantitation results

As can be seen in Table S8A,B, all variation coefficients fall within the 20% limit and were ≤5.0 WBC/µL for body fluids. By using BF XN check, the lower limit of quantitation (LoQ) for WBC was defined as 5.0 cells/μL and for RBC as 2.0 × 10^3^/μL.

## 4 DISCUSSION

The XN‐350 is the smallest of the XN‐L series and its compact presentation makes it an essential tool that fits the needs of (satellite) laboratories in specialized outpatient centers, for example, pediatrics, oncology, or dialysis, with a lower throughput of samples. Due to the low required sample volume of only 25 µL,^18^ this analyzer can be used if quick tests are needed for a more optimized patient journey and to eliminate unnecessary send outs saving both time and money.

Overall the performance results of the XN‐350 were consistent and accurate as compared to the XN‐1000. Method comparison between XN‐350 and XN‐1000 shows comparable results for both the whole blood as well as body fluid modes. Although all results met the acceptance criteria, method comparison results show a number of statistically significant proportional and systemic biases; however, these are mostly likely due to analytical differences and are not clinically significant. For example, MCH of samples included in the studies showed bias only in the Bland‐Altman analysis between instruments; however, the bias is not clinically significant as no proportional bias was shown. Any biases present are not to the extent that the course of treatment for patients would be affected. Bias may be due to inter‐instrument differences as shown in method comparison data previously published^19^ and is present on several calculated parameters such as MCH and MCHC, but is not clinically significant. We observed a low correlation in basophil counts between the two analyzers (*r*_s_ = 0.769). This low correlation may have been due to statistical uncertainty as a result of low basophil numbers, as not many samples were included with high numbers of basophils. Low correlation values in basophil counts have been previously published for the XN analysers.^4^

Both PLT‐I and PLT‐O results were evaluated in method comparison and compared well between XN350 and XN1000 for the whole measuring range. PLT‐I is the initial measurement taken, but in case of interference in the impedance count, reflex measurement in a different channel is done. For the XN‐350, this is the PLT‐O count in the RET channel, but on the XN‐1000 it is PLT‐F. The platelet measurement in the PLT‐F channel provides the most accurate and precise automated PLT count of the three.^4^, ^20^

Flagging accuracy for both the XN‐350 and XN‐1000 was compared to manual differential for abnormal cell types and the same agreement in the frequency of true flag messages of the XN‐350 compared to the XN‐1000 was observed. Both analyzers seem to accurately flag samples at approximately the same amount. Therefore, it can be concluded that the two systems are comparable for the parameters tested. It also should be acknowledged that the XN‐350 has a combined blast/abnormal lymphocyte flag; therefore, the flagging results of the two cell types were combined. Briggs et al. evaluated the flagging performance of the XN‐1000 for blasts, abnormal lymphocytes, and atypical lymphocytes for the first time and their results were similar to ours. In our study, we had a higher sensitivity for abnormal lymphocytes (90.5% vs 37.5%) on the XN‐1000, although it is a difficult comparison as the flagging results for abnormal lymphocytes and blasts were combined in our study and not in Briggs et al.^4^ Specificity for atypical lymphocytes was similar in both studies and very high; however, in our study, sensitivity was quite low on both the XN‐350 and XN‐1000. This may be due to the discrepancies as to how atypical lymphocytes are defined as there are no standardized definitions. Trained laboratory technologists overestimate the presence of atypical lymphocytes and additional clinical information could lead to a better interpretation of blood cell morphology.^21^ Overestimation could have occurred in this study as well as to whether lymphocytes are in fact atypical. Variation may be seen as well in flagging due to the thresholds established for positivity.

In the case of repeatability and reproducibility, the measurements were carried out in the pre‐diluted whole blood, and body fluid modes. For whole blood in the repeatability test, the measurements had a slightly smaller standard deviation and variance coefficient compared with the measurements in pre‐diluted mode. Although this difference is not very large, it may have to do with manual error from pipetting the dilutions. In the pre‐diluted mode, the blood samples are naturally diluted 1:7 with DCL Cellpack. In addition, the XN‐350 did indeed give a repeatability problem in whole blood mode with the basophils and the eosinophils due to the low cell numbers, which was expected. Variability was also seen in some body fluid types, especially #PMN, which was also due to the high statistical variation as a result of the very low WBC in the body fluid samples. It can be concluded, however, that the Sysmex XN‐350 performs repeatable and reproducible measurements on the same device under the same conditions for the tested parameters for whole blood and body fluids.

In carryover for whole blood, during the measurement of the PLT, PLT‐I was chosen because the Sysmex XN‐350 does not have a channel that can measure the PLT‐F parameter and PLT‐O is officially not available as a diagnostic parameter on the XN‐1000. Overall the carryover ratios for all parameters in whole blood and body fluid mode were within the 0.5% acceptance criteria indicating the results were acceptable. A limitation of this study was the manual manipulation of the RBC and HGB for whole blood. Ideally, these samples would have occurred naturally, but the results were acceptable. In addition, the LoQ of the XN‐350 in terms of CSF is comparable to that of the XN‐1000 at ≤5.0 WBC/µL and for RBC at 2.0 × 10^3^/µL, which is better than most other body fluid analyzers on the market.^6^

For most of the parameters, the stability remains 8h after blood sampling, which is the preferred maximum time elapsed after venipuncture before routine processing. All evaluated parameters, except MCV, are stable 12 hours after sampling when stored at room temperature. It is known that MCV changes more rapidly than other parameters over time,^22^ but based on the study results MCV should be measured within 4h, which is acceptable especially for smaller laboratory settings as there is less time between venipuncture and processing. This emphasizes the importance of refrigerating samples within 4 hours of venipuncture for most accurate results, especially when further RBC parameter tests may be needed.

The aim of this study was to evaluate the performance of the XN‐350 analyzer against XN‐1000 and evaluate the interchangeability of these analyzers in terms of quality of the results as it meets the needs of niche laboratories. The compactness makes it suitable for remote, specialty laboratories in areas such as pediatrics, neurology, and oncology to eliminate the need for additional send outs and waiting time. The accurate leukocyte count, particularly neutrophils, makes it a reliable tool for chemotherapy monitoring in oncology clinics. Not only are the whole blood features and measurements comparable to that of the much larger, XN‐1000 analyzer, it also has the ability to process body fluids, which is essential for use in dialysis clinics and neurology clinics in an accurate and convenient way. In conclusion, this study shows good performance results for the XN‐350 analyzer and should be adopted as reliable and comparable to the XN‐1000 analyzer.

## CONFLICT OF INTEREST

The authors have no competing interests.

## Supporting information

Fig S1

Fig S2

Table S1‐S8
