# Supplementary figures and images for: The Sysmex XN‐L (XN‐350) hematology analyzer offers a compact solution for laboratories in niche diagnostics
Source: Int J Lab Hematol. 2020 Sep 19;43(1):29–39. doi: 10.1111/ijlh.13339 (PMC7891428; doi:10.1111/ijlh.13339)

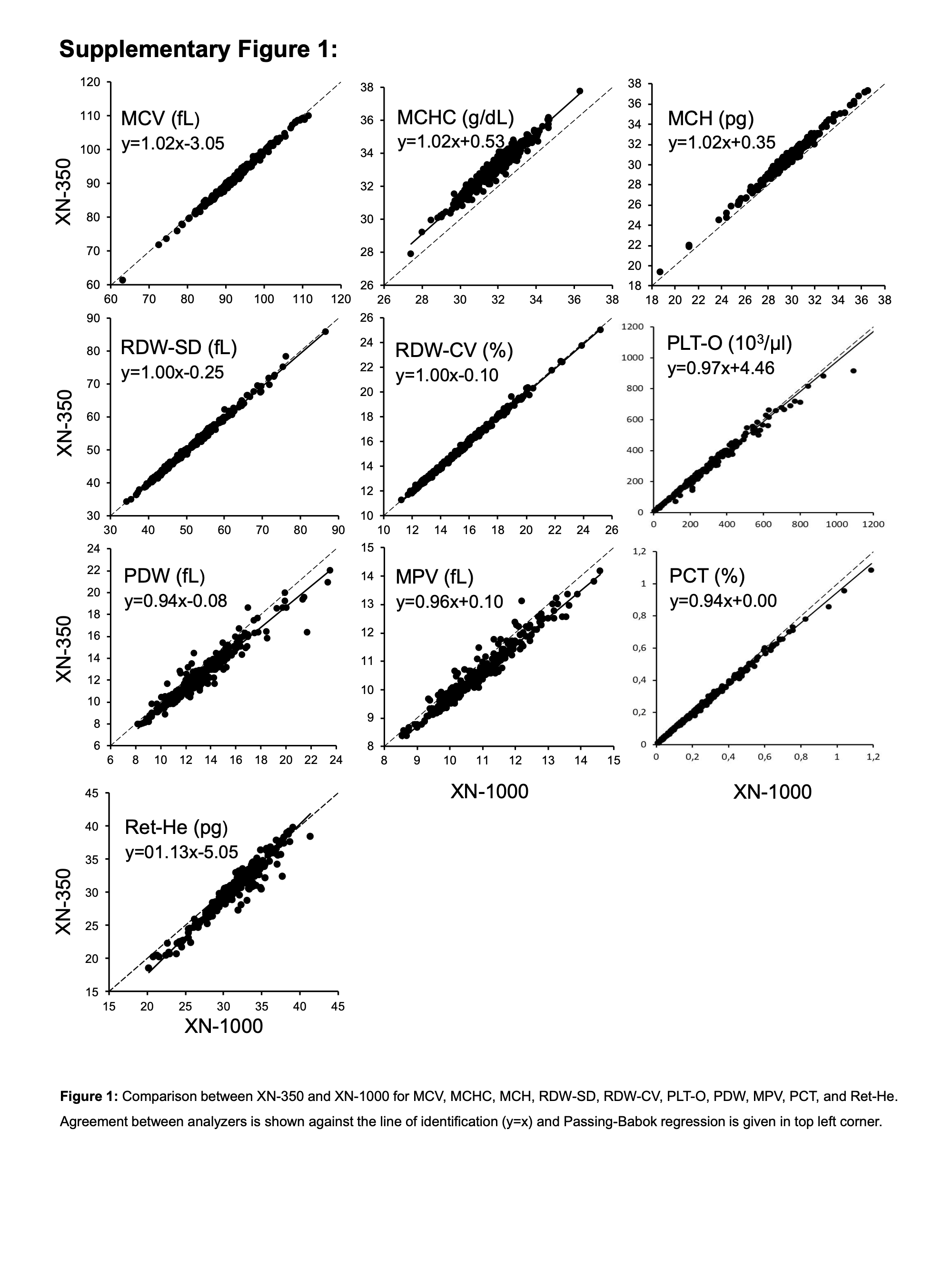

Supplement: Supplementary file 1 — Fig S1 [file IJLH-43-29-s001.jpg]

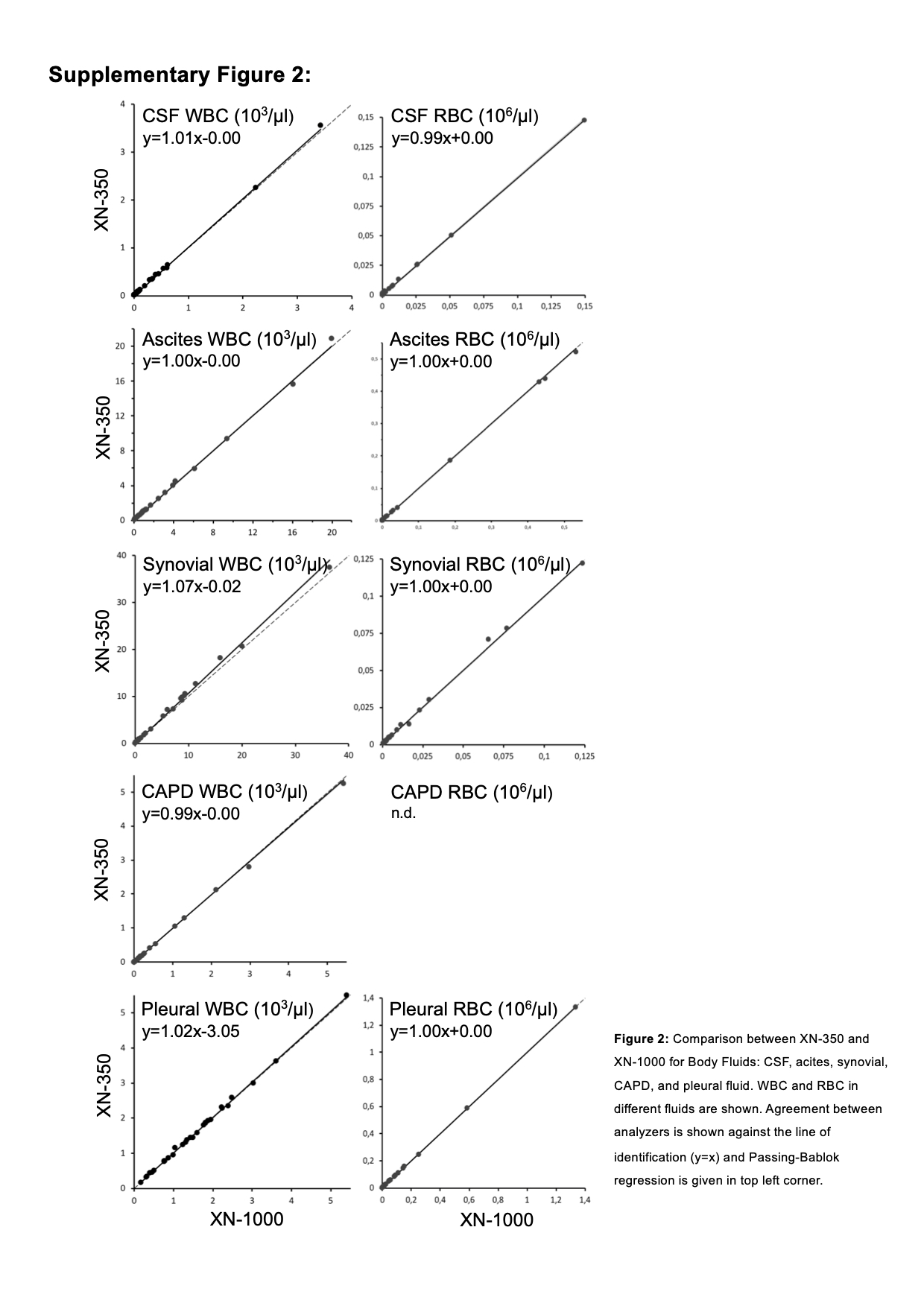

Supplement: Supplementary file 2 — Fig S2 [file IJLH-43-29-s002.jpg]
