# Supplementary material for: The Sysmex XN‐L (XN‐350) hematology analyzer offers a compact solution for laboratories in niche diagnostics
Source: Int J Lab Hematol. 2020 Sep 19;43(1):29–39. doi: 10.1111/ijlh.13339 (PMC7891428; doi:10.1111/ijlh.13339)
Supplement: Supplementary file 3 — Table S1‐S8 [file IJLH-43-29-s003.docx]

**Supplementary Tables**

**Supplementary Table 1:** Repeatability of Whole Blood Samples in Whole Blood and Pre-Dilution Modes on the XN-350

| **Parameter** | **Whole blood mode** | | **Pre-diluted mode** | |
| --- | --- | --- | --- | --- |
|  | **%CV** | **Claim Sysmex (%)** | **%CV** | **Claim Sysmex (%)** |
| WBC | 1.0 | <3 | 1.5 | <5.0 |
| RBC | 1.0 | <1.5 | 1.1 | <4.5 |
| HGB | 0.9 | <1.5 | 0.8 | <4.5 |
| HCT | 1.0 | <1.5 | 1.0 | <4.5 |
| MCV | 1.2 | <1.5 | 0.5 | <4.5 |
| MCH | 0.8 | <2.0 | 1.1 | <4.5 |
| MCHC | 1.4 | <2.0 | 1.1 | <6.0 |
| RDW-SD | 1.8 | <3.0 | 1.3 | <6.0 |
| RDW-CV | 0.6 | <3.0 | 0.9 | <6.0 |
| Reticulocytes (#) | 5.0 | <15 | 5.2 | <35 |
| Ret-He | 1.3 | <5.0 | 5.0 | <5.0 |
| Neutrophils (#) | 1.0 | <8.0 | 1.7 | <16.0 |
| Lymphocytes (#) | 6.4 | <8.0 | 5.5 | <16.0 |
| Monocytes (#) | 5.0 | <20 | 4.6 | <40 |
| Eosinophils (#) | 13.2 | <25 | 11 | <40 |
| Basophils (#) | 17.4 | <40 | 22.9 | <50 |
| Immature granulocytes (#) | 22 | <25 | 31 | <75 |
| PLT | 3.4 | <4.0 | 5.8 | <12.0 |
| PCT | 2.5 | <6.0 | 9.3 | <12.0 |
| %CV, coefficient of variation percentage. | | | | |

**Supplementary Table 2:** Whole Blood Mode Reproducibility for Quality Control XN CHECK Levels 1, 2 and 3.

|  | **XN CHECK Level 1** | | | | **XN CHECK Level 2** | | | | **XN CHECK Level 3** | | | |
| --- | --- | --- | --- | --- | --- | --- | --- | --- | --- | --- | --- | --- |
| Parameter | MEAN | SD | %CV | Claim Sysmex (%CV) | MEAN | SD | %CV | Claim Sysmex (%CV) | MEAN | SD | %CV | Claim Sysmex (%CV) |
| WBC(10^3^/ul) | 2.43 | 0.07 | 2.95 | <10 | 6.86 | 0.13 | 1.92 | <9 | 15.7 | 0.26 | 1.67 | <9 |
| RBC(10^6^/ul) | 2.33 | 0.02 | 1.01 | <5 | 4.40 | 0.03 | 0.75 | <5 | 5.35 | 0.03 | 0.64 | <5 |
| HGB(g/dl) | 6.00 | 0.07 | 1.21 | <4 | 12.73 | 0.08 | 0.62 | <4 | 16.6 | 0.11 | 0.69 | <4 |
| HCT(%) | 17.8 | 0.18 | 1.03 | <10 | 36.8 | 0.33 | 0.91 | <10 | 46.6 | 0.40 | 0.86 | <10 |
| MCV(fl) | 76.4 | 0.45 | 0.59 | <5 | 83.5 | 0.34 | 0.41 | <5 | 87.0 | 0.38 | 0.44 | <5 |
| MCH(Pg) | 25.8 | 0.32 | 1.25 | <9 | 28.9 | 0.24 | 0.84 | <8 | 31.0 | 0.22 | 0.70 | <8 |
| MCHC(g/dl) | 33.8 | 0.49 | 1.46 | <15 | 34.7 | 0.35 | 1.00 | <14 | 35.6 | 0.34 | 0.94 | <14 |
| PLT(10^3^/ul) | 61.1 | 3.89 | 6.38 | <80 | 234.4 | 8.81 | 3.76 | <15 | 555.7 | 8.77 | 1.58 | <9 |
| RDW-SD(fl) | 46.1 | 0.39 | 0.85 | <10 | 44.5 | 0.27 | 0.61 | <10 | 41.9 | 0.30 | 0.73 | <10 |
| RDW-CV(%) | 16.7 | 0.14 | 0.83 | <10 | 14.6 | 0.11 | 0.74 | <10 | 13.08 | 0.06 | 0.45 | <10 |
| PCT(%) | 0.07 | 0.01 | 9.53 | <116 | 0.23 | 0.01 | 4.55 | <30 | 0.54 | 0.01 | 1.86 | <25 |
| NEUT#(10^3^/ul) | 1.72 | 0.05 | 3.14 | <20 | 5.21 | 0.11 | 2.13 | <15 | 12.67 | 0.25 | 1.95 | <15 |
| LYMPH#(10^3^/ul) | 0.62 | 0.05 | 8.65 | <40 | 1.52 | 0.08 | 5.12 | <20 | 2.77 | 0.11 | 3.95 | <20 |
| MONO#(10^3^/ul) | 0.09 | 0.04 | 45.55 | <80 | 0.13 | 0.05 | 42.06 | <60 | 0.30 | 0.07 | 23.63 | <50 |
| EO#(10^3^/ul) | 0.00 | 0.00 | 0.00 | <50 | 0.00 | 0.00 | 0.00 | <50 | 0.00 | 0.00 | 0.00 | <50 |
| BASO#(10^3^/ul) | 0.00 | 0.00 | 0.00 | <78 | 0.00 | 0.00 | 0.00 | <78 | 0.00 | 0.00 | 227.43 | <78 |
| IG#(10^3^/ul) | 0.07 | 0.03 | 46.82 | <30 | 0.11 | 0.02 | 16.92 | <30 | 0.25 | 0.03 | 13.73 | <25 |
| RET#(10^6^/ul) | 0.19 | 0.01 | 2.79 | <30 | 0.14 | 0.00 | 2.46 | <30 | 0.08 | 0.00 | 4.14 | <30 |
| RET-HE(Pg) | 22.0 | 0.13 | 0.60 | <10 | 23.1 | 0.13 | 0.57 | <10 | 24.7 | 0.29 | 1.16 | <10 |
| SD, standard deviation; %CV, coefficient of variation percentage. | | | | | | | | | | | | |

**Supplementary Table 3:** Body Fluid Reproducibility for Quality Control XN CHECK BF Levels 1 and 2.

|  | **XN CHECK BF L1** | | | | **XN CHECK BF L2** | | | |
| --- | --- | --- | --- | --- | --- | --- | --- | --- |
| Parameter | MEAN | SD | %CV | Claim Sysmex (%CV) | MEAN | SD | %CV | Claim Sysmex (%CV) |
| WBC-BF (x10^3^/μl) | 0.08 | 0.00 | 5.24 | ≤ 35 | 0.33 | 0.02 | 5.23 | ≤ 25 |
| RBC-BF (x10^6^/μl) | 0.03 | 0.00 | 4.08 | ≤ 35 | 0.08 | 0.00 | 3.32 | ≤ 25 |
| MN# (x10^3^/μl) | 0.03 | 0.00 | 11.32 | ≤ 70 | 0.12 | 0.01 | 5.69 | ≤ 60 |
| PMN# (x10^3^/μl) | 0.05 | 0.00 | 5.02 | ≤ 70 | 0.21 | 0.01 | 5.52 | ≤ 60 |
| TC-BF# (x10^3^/μl) | 0.08 | 0.00 | 5.24 | ≤ 35 | 0.33 | 0.02 | 5.23 | ≤ 25% |
| BF, body fluid mode; PMN, polymorphonuclear leukocytes; MN, mononuclear leukocytes; TC-BF, total cell count body fluid mode; SD, standard deviation; %CV, coefficient of variation percentage. | | | | | | | | |

**Supplementary Table 4A:** Repeatability of CAPD Fluid on the XN-350 in Body Fluid Mode.

| **CAPD** |  |  |  |
| --- | --- | --- | --- |
|  | Mean of 10 runs | SD | %CV |
| WBC-BF (x10^3^/μl) | 0.0414 | 0.00246 | 5.94 |
| RBC-BF (x10^6^/μl) | 0 | 0 | 0 |
| MN# (x10^3^/μl) | 0.0331 | 0.00166 | 5.03 |
| PMN# (x10^3^/μl) | 0.00830 | 0.00149 | 18.0 |
| TC-BF# (x10^3^/μl) | 0.0425 | 0.00250 | 5.90 |
| BF, body fluid mode; PMN, polymorphonuclear leukocytes; MN, mononuclear leukocytes; TC-BF, total cell count body fluid mode; SD, standard deviation; %CV, coefficient of variation percentage. | | | |

**Supplementary Table 4B:** Repeatability of Ascites Fluid on the XN-350 in Body Fluid Mode

| **Ascites** |  |  |  |
| --- | --- | --- | --- |
|  | Mean of 10 runs | SD | %CV |
| WBC-BF (x10^3^/μl) | 0.131 | 0.00401 | 3.06 |
| RBC-BF (x10^6^/μl) | 0.000300 | 0.000483 | 0 |
| MN# (x10^3^/μl) | 0.111 | 0.00359 | 3.24 |
| PMN# (x10^3^/μl) | 0.0204 | 0.00514 | 25.2 |
| TC-BF# (x10^3^/μl) | 0.180 | 0.00392 | 2.18 |
| BF, body fluid mode; PMN, polymorphonuclear leukocytes; MN, mononuclear leukocytes; TC-BF, total cell count body fluid mode; SD, standard deviation; %CV, coefficient of variation percentage. | | | |

**Supplementary Table 4C:** Repeatability of Synovial Fluid on the XN-350 in Body Fluid Mode

| **Synovial** |  |  |  |
| --- | --- | --- | --- |
|  | Mean of 10 runs | SD | %CV |
| WBC-BF (x10^3^/μl) | 0.0122 | 0.00274 | 22.5 |
| RBC-BF (x10^6^/μl) | 0.0713 | 0.00226 | 0 |
| MN# (x10^3^/μl) | 0.00530 | 0.000823 | 15.5 |
| PMN# (x10^3^/μl) | 0.00690 | 0.00247 | 35.8 |
| TC-BF# (x10^3^/μl) | 0.0141 | 0.00292 | 20.7 |
| BF, body fluid mode; PMN, polymorphonuclear leukocytes; MN, mononuclear leukocytes; TC-BF, total cell count body fluid mode; SD, standard deviation; %CV, coefficient of variation percentage. | | | |

**Supplementary Table 4D:** Repeatability of Pleural Fluid on the XN-350 in Body Fluid Mode

| **Pleural** |  |  |  |
| --- | --- | --- | --- |
|  | Mean of 10 runs | SD | %CV |
| WBC-BF (x10^3^/μl) | 0.422 | 0.0145 | 3.43 |
| RBC-BF (x10^6^/μl) | 0.0482 | 0.00148 | 0 |
| MN# (x10^3^/μl) | 0.102 | 0.00398 | 3.91 |
| PMN# (x10^3^/μl) | 0.320 | 0.0114 | 3.57 |
| TC-BF# (x10^3^/μl) | 0.436 | 0.0144 | 3.31 |
| BF, body fluid mode; PMN, polymorphonuclear leukocytes; MN, mononuclear leukocytes; TC-BF, total cell count body fluid mode; SD, standard deviation; %CV, coefficient of variation percentage. | | | |

**Supplementary Table 4E:** Repeatability of CSF with WBC <4/ul on the XN-350 in Body Fluid Mode

| **CSF WBC <4/μl** |  |  |  |
| --- | --- | --- | --- |
|  | Mean of 10 runs | SD | %CV |
| WBC-BF (x10^3^/ul) | 0.00470 | 0.000674 | 14.4 |
| RBC-BF (x10^6^/ul) | 0 | 0 | 0 |
| MN# (x10^3^/ul)* | 0.00450 | 0.000755 | 16.8 |
| PMN# (x10^3^/ul)* | 0.000250 | 0.000462 | 185 |
| TC-BF# (x10^3^/μl) | 0.00480 | 0.000788 | 16.4 |
| *Only 8 results due to processing error.  BF, body fluid mode; PMN, polymorphonuclear leukocytes; MN, mononuclear leukocytes; TC-BF, total cell count body fluid mode; SD, standard deviation; %CV, coefficient of variation percentage. | | | |

**Supplementary Table 4F:** Repeatability of CSF with WBC ±10/uL on the XN-350 in Body Fluid Mode

| **CSF WBC ±10/μl** |  |  |  |
| --- | --- | --- | --- |
|  | Mean of 10 runs | SD | %CV |
| WBC-BF (x10^3^/μl) | 0.00820 | 0.00168 | 20.6 |
| RBC-BF (x10^6^/μl) | 0 | 0 | 0 |
| MN# (x10^3^/μl) | 0.00760 | 0.00177 | 23.4 |
| PMN# (x10^3^/μl) | 0.000600 | 0.000516 | 86.1 |
| TC-BF# (x10^3^/μl) | 0.00860 | 0.00157 | 18.3 |
| BF, body fluid mode; PMN, polymorphonuclear leukocytes; MN, mononuclear leukocytes; TC-BF, total cell count body fluid mode; SD, standard deviation; %CV, coefficient of variation percentage. | | | |

**Supplementary Table 4G:** Repeatability of CSF with WBC ±50/μl on the XN-350 in Body Fluid Mode

| **CSF WBC ±50/μl** |  |  |  |
| --- | --- | --- | --- |
|  | Mean of 9* runs | SD | %CV |
| WBC-BF (x10^3^/μl) | 0.0594 | 0.00482 | 8.12 |
| RBC-BF (x10^6^/μl) | 0 | 0 | 0 |
| MN# (x10^3^/μl) | 0.0234 | 0.00245 | 10.5 |
| PMN# (x10^3^/μl) | 0.0360 | 0.00282 | 7.86 |
| TC-BF# (x10^3^/μl) | 0.0598 | 0.00468 | 7.84 |
| *Run 10 was not able to be processed due to low volume.  BF, body fluid mode; PMN, polymorphonuclear leukocytes; MN, mononuclear leukocytes; TC-BF, total cell count body fluid mode; SD, standard deviation; %CV, coefficient of variation percentage. | | | |

**Supplementary Table 5:** XN-350 Sysmex Reproducibility Claim for Body Fluids

| WBC Range | Acceptable %CV |
| --- | --- |
| 5 - 15 x10^3^/μl | CV <30.0 |
| 16 - 30 x10^3^/μl | CV <20.0 |
| 31 - 50 x10^3^/μl | CV<15.0 |
| %CV=coefficient of variation percentage | |

**Supplementary Table 6:** Whole Blood Carryover

| Parameter | Criteria | | Selected samples | | Carryover ratio (%) |
| --- | --- | --- | --- | --- | --- |
|  | HTV | LTV | HTV | LTV |  |
| WBC (x10^3^/μl) | >90.0 | >0 en <3.0 | 93.11 | 1.94 | 0.03 |
| RBC (x10^6^/μl) | >6.20 | >0 en <1.5 | 8.19 | 1.15 | 0.29 |
| HGB (g/dl) | >22.0 | >0 en <5.0 | 22.6 | 3.38 | 0.00 |
| PLT (x10^3^/μl)) | >990 | >0 en <30 | 995 | 22.0 | 0.20 |
| HTV=high-target value; processed three times (H1, H2, H3) LTV= low-target value, processed three times (B1, B2, B3) | | | | | |

**Supplementary Table 7:** WBC and RBC Carryover Result Measurements for CSF in Body Fluid Mode

| Parameter | Criteria | | Selected samples | | Carryover ratio (%) |
| --- | --- | --- | --- | --- | --- |
|  | HTV | LTV | HTV | LTV |  |
| WBC-BF | ≥10,000/µl | 0.0/µl | 20.440 x10^3^/µl | 0.001 x10^3^/µl | 0.0049 |
| RBC-BF | OR 100,000/µl |  | 0.02 x10^6^/µl | 0.00 x10^6^/µl | 0.00 |
| HTV=high-target value; processed three times (H1, H2, H3) LTV= low-target value, processed three times (B1, B2, B3) | | | | | |

| **Supplementary Table 8A:** Limit of Quantification for WBC in Body Fluid Mode | | | | | | | | | | |
| --- | --- | --- | --- | --- | --- | --- | --- | --- | --- | --- |
|  | **Run 1** | **Run 2** | **Run 3** | **Run 4** | **Run 5** |  |  |  |  |  |
| **Sample** | **WBC/μl** | **WBC/μl** | **WBC/μl** | **WBC/μl** | **WBC/μl** | **Mean** | **SD** | **CV%** |  |  |
| 1 | 2.0 | 2.0 | 2.0 | 2.0 | 2.0 | 2.0 | 0.00 | 0.0 |  |  |
| 2 | 2.0 | 3.0 | 3.0 | 3.0 | 3.0 | 2.8 | 0.45 | 16.0 |  |  |
| 3 | 1.0 | 1.0 | 1.0 | 1.0 | 1.0 | 1.0 | 0.00 | 0.0 |  |  |
| 4 | 1.0 | 1.0 | 1.0 | 1.0 | 1.0 | 1.0 | 0.00 | 0.0 |  |  |
| 5 | 5.0 | 5.0 | 5.0 | 4.0 | 4.0 | 4.6 | 0.55 | 11.9 |  |  |
| 6 | 0.0 | 0.0 | 0.0 | 0.0 | 0.0 | 0.0 | 0.00 | 0.0 |  |  |
| SD, standard deviation; %CV=coefficient of variation percentage | | | | | | | | |  |  |
| **Supplementary Table 8B:** Limit of Quantification for RBC in Body Fluid Mode | | | | | | | | | | |
|  | **Run 1** | **Run 2** | **Run 3** | **Run 4** | **Run 5** |  |  |  |  |  |
| **Sample** | **RBC x10^3^/μl** | **RBC x10^3^/μl** | **RBC x10^3^/μl** | **RBC x10^3^/μl** | **RBC x10^3^/μl** | **Mean** | **SD** | **CV%** |  |  |
| 1 | 0.0 | 0.0 | 0.0 | 0.0 | 0.0 | 0.0 | 0.0 | 0.0 |  |  |
| 2 | 0.0 | 0.0 | 0.0 | 0.0 | 0.0 | 0.0 | 0.0 | 0.0 |  |  |
| 3 | 0.0 | 0.0 | 0.0 | 0.0 | 0.0 | 0.0 | 0.0 | 0.0 |  |  |
| 4 | 0.0 | 0.0 | 0.0 | 0.0 | 0.0 | 0.0 | 0.0 | 0.0 |  |  |
| 5 | 0.0 | 0.0 | 0.0 | 0.0 | 0.0 | 0.0 | 0.0 | 0.0 |  |  |
| 6 | 2.0 | 2.0 | 2.0 | 2.0 | 2.0 | 2.0 | 0.0 | 0.0 |  |  |
| SD=standard deviation, %CV=coefficient of variation percentage | | | | | | | | |  |  |
